# Retraction: PknE, a Serine/Threonine Protein Kinase of *Mycobacterium tuberculosis* Initiates Survival Crosstalk That Also Impacts HIV Coinfection

**DOI:** 10.1371/journal.pone.0211247

**Published:** 2019-01-17

**Authors:** The PLOS ONE Editors

Following publication of this article [1], a number of concerns regarding Western blot images in Figs 1 and 3 were raised to the attention of the *PLOS ONE* Editors. Specific concerns include similarities between bands in the following panels:

- Fig 1A Ctrl 0’ and CΔE 0’
- Fig 1E Ctrl 0’, Rv 0’, ΔE 0’ and CΔE 0’
- Fig 1I LPS 0’, Rv 0’, and ΔE 0’
- Fig 1I Ctrl 60’ and LPS 60’
- Fig 1I Pan SAPK/JNK 0’ and Pan SAPK/JNK 60’
- Fig 1C Pan ERK 1/2 Ctrl and Fig 3B Total Erk Ctrl
- Fig 1C Pan ERK 1/2 LPS and Fig 3B Total Erk DMSO and CΔE
- Fig 1C Pan ERK 1/2 Rv and Fig 3B Total Erk LPS
- Fig 1C Pan ERK 1/2 ΔpknE and Fig 3B Rv
- Fig 1C Pan ERK 1/2 CΔE and Fig 3B ΔpknE
- Fig 3A Ctrl and DMSO

The authors have provided the full blots underlying the panels in which possible duplications were observed; however, the raw uncropped blots underlying the other Western blot time points in Figs 1 and 3 are now unavailable.

The authors acknowledge duplication between bands shown in the Fig 3B Total Erk panel and those shown in Fig 1C Pan Erk 1/2, and indicate this arose due to an unintentional error in image selection for Fig 3. The authors stand by the accuracy of the underlying findings and other figure panels presented in the article.

The *PLOS ONE* Editors have assessed the available data and files, and have consulted with a member of the Editorial Board. While the concerns regarding Fig 3B Total Erk were addressed in follow up, concerns remain that there are discrepancies between other figure panels and the uncropped blot images provided by the authors.

Additional issues were raised regarding the methodology and interpretation of the data. Specifically,

- It is unclear whether loading control blots were carried out for all experiments/time points.
- It is unclear how the Western blot data were quantified and what steps were taken in data normalization.
- Separate Western blots were run for each time point in the time course experiments shown in Fig 1, raising concerns about the validity of comparisons made between signaling responses at different timepoints.
- Some data points in Fig 2 are accompanied by a *, indicating p<0.05 by two-way ANOVA. The figure legend states “The results from three independent experiments are shown. The O.D values denote standard error of the means”; however, there are no error bars, and the data used for the statistical analysis are unclear.
- In Fig 4, some of the statistically significant differences have very small changes in value, and it is difficult to assess whether this is biologically significant.

In light of these concerns, which call into question the integrity and reliability of the published data, the *PLOS ONE* Editors retract this article.

DKP, LEH, SN did not agree with retraction.
